# Serotonin syndrome after an overdose of over‐the‐counter medicine containing dextromethorphan

**DOI:** 10.1002/jgf2.469

**Published:** 2021-06-04

**Authors:** Nina Omoto, Yohei Kanzawa, Naoto Ishimaru, Saori Kinami

**Affiliations:** ^1^ Department of General Internal Medicine Akashi Medical Center Hyogo Japan

**Keywords:** dextromethorphan, over‐the‐counter medicine, serotonin syndrome

## Abstract

Serotonin syndrome is a potentially life‐threatening adverse reaction from therapeutic drug use, intentional self‐poisoning, or inadvertent interactions between drugs. We report a case of the serotonin syndrome after an overdose of a commonly available over‐the‐counter cough medicine, *Shin CONTAC sekidome daburu jizokusei*. Over‐the‐counter drugs containing dextromethorphan may, in rare cases, cause health problems requiring hospitalization or worse. An appropriate explanation from the pharmacist at the time of purchase, although not mandatory, is recommended.

## 1 INTRODUCTION

Serotonin syndrome is a potentially life‐threatening adverse drug reaction resulting from therapeutic drug use, intentional self‐poisoning, or inadvertent interactions between drugs. We report a case of the serotonin syndrome after an overdose of a commonly available over‐the‐counter medicine.

## 2 PRESENTATION

A 23‐year‐old woman taking mirtazapine with a history of mood disorder with depressive features and an adjustment disorder presented to the emergency department with disturbance of consciousness beginning on the previous day. She had not eaten anything for three days before admission. Twenty‐seven hours before her visit, she took 30 tablets of an over‐the‐counter cough medicine, *Shin CONTAC sekidome daburu jizokusei*, containing 1800 mg of dextromethorphan and 6000 mg of diprophylline. The recommended limit is two capsules per day. She had not taken an overdose of her usual prescription of mirtazapine. Five hours before admission, she was found semiconscious in her apartment. On examination, consciousness was Glasgow coma scale E3V4M4, blood pressure was 125/74 mmHg, heart rate 135 beats per minute, respiratory rate 24 breaths per minute, temperature 37.8℃, and oxygen saturation was 95% while the patient was breathing ambient air. The patient met the Hunter serotonin toxicity criteria; she was markedly sweaty, had ocular clonus, and unstimulated clonus in her extremities.^1^ Rigidity, indicative of malignant syndrome and malignant fever, was absent. Bilateral pupils were 3.5 mm, pupillary light reflex was prompt, and physical examination showing damp skin without flushing did not indicate anticholinergic poisoning. Only morphine was positive on comprehensive toxicologic screening (Triage DOA), which indicated false‐positive reaction by dextromethorphan. Regular medications (mirtazapine 15 mg, chlorpromazine 50 mg, promethazine 25 mg, suvorexant 20 mg, and zopiclone 10 mg) were adjourned, and cyproheptadine 24 mg/day was administered to treat serotonin syndrome until the fifth day of hospitalization. Lumbar puncture was considered to rule out meningitis and encephalitis, but it was not performed because her consciousness improved so rapidly after initial treatment. On the second day of hospitalization, the patient regained consciousness and the clonus disappeared. She was discharged on the fifth day of hospitalization, and regular medication was resumed.

## 3 DISCUSSION

Our patient had serotonin syndrome after an overdose of a commonly available over‐the‐counter cough medication. Serotonin syndrome is classically associated with the simultaneous administration of two serotonergic agents.^2^ A striking number of drugs and drug combinations have been associated with the serotonin syndrome, including monoamine oxidase inhibitors, tricyclic antidepressants, SSRIs, opiate analgesics, over‐the‐counter cough medicines, antibiotics, weight‐reduction agents, antiemetics, anti‐migraine agents, illegal street drugs, and herbal products.^2^

Our patient regularly used mirtazapine on prescription, a noradrenergic and specific serotonergic antidepressant. Mirtazapine works by its central presynaptic alpha‐2 adrenergic antagonist effects, resulting in increased release of norepinephrine and serotonin.^3^ It is also a potent antagonist of 5‐HT_2_ and 5‐HT_3_ serotonin receptors and of H1 histamine receptors, and it is a moderate peripheral alpha‐1 adrenergic and muscarinic antagonist; it does not inhibit the reuptake of norepinephrine or serotonin.^3^

Dextromethorphan, the constituent medicine which our patient overdosed on, is a commonly used antitussive drug that decreases the sensitivity of cough receptors and interrupts cough impulse transmission by depressing the medullary cough center through sigma receptor stimulation.^4^ It also inhibits the reuptake of serotonin.^5^

No single receptors appear to be responsible for the development of the serotonin syndrome, although several lines of evidence converge to suggest that agonism of 5‐HT_2a_ receptors contributes substantially to the condition.^2^

In this case, the patient was taking mirtazapine regularly, which increased the release of serotonin. In addition to the intake of mirtazapine, we suggest that consumption of 30 tablets of dextromethorphan would have led to serotonin syndrome. We suggest that the effect may have been enhanced by the patient's history of taking mirtazapine. The overdose of dextromethorphan may have caused a further increase in serotonin concentration due to inhibition of serotonin reuptake, mainly due to enhanced stimulation of 5‐HT_1A_ receptors, resulting in the serotonin syndrome. In addition, the patient had not eaten for three days before hospitalization and she had renal impairment due to dehydration, which may have led to delayed excretion of dextromethorphan. The overdose may have induced a decrease in the detoxification efficiency of the patient's hepatic and renal metabolism, resulting in her regular medications also having the potential to induce serotonin syndrome.

The patient developed serotonin syndrome as a result of an overdose of an over‐the‐counter medication, which contains dextromethorphan. In the United States, recreational use of over‐the‐counter drugs containing dextromethorphan has long been a problem among young people and a case of serotonin syndrome induced by Coricidin HBP, which also contains dextromethorphan, has been reported.^4^ Serotonin syndrome from another case of overdose of over‐the‐counter medication containing dextromethorphan has been reported; a patient was regularly taking serotonin‐norepinephrine reuptake inhibitor.^6^

In Japan, there have been no such reports of serotonin syndrome being triggered by over‐the‐counter drugs. Our case of serotonin syndrome triggered by an overdose of an over‐the‐counter drug is therefore the first to be reported, but unlikely to be the first actual case. These over‐the‐counter drugs containing dextromethorphan are sold as second‐class over‐the‐counter drugs in Japan, defined as those containing ingredients that in rare cases may cause health problems requiring hospitalization or worse. An appropriate explanation from the pharmacist at the time of purchase is recommended, although not mandatory. Primary physicians should also take care of the risk of serotonin syndrome. For immunocompetent adult outpatients with cough due to acute bronchitis, no routine medication is recommended.^7^ When patients with risk of serotonin syndrome need treatment for coughs, primary physicians should reconsider routine prescription of medications and to search for the cause of the cough and consider treatment according to specific diagnosis.^8^

## CONFLICTS OF INTEREST

The authors declare no conflicts of interest in relation to this article.

## INFORMED CONSENT

The patient agreed to the publication of her case by providing informed consent.

## References

[1] Ables AZ , Nagubilli R . Prevention, recognition, and management of serotonin syndrome. Am Fam Physician. 2010;81(9):1139–42. 20433130

[2] Boyer EW , Shannon M . The serotonin syndrome. N Engl J Med. 2005;352(11):1112–20. 15784664 10.1056/NEJMra041867

[3] Hernández JL , Ramos FJ , Infante J , Rebollo M , González‐Macías J . Severe serotonin syndrome induced by mirtazapine monotherapy. Ann Pharmacother. 2002;36(4):641–3. 11918514 10.1345/aph.1A302

[4] Ganetsky M , Babu KM , Boyer EW . Serotonin syndrome in dextromethorphan ingestion responsive to propofol therapy. Pediatr Emerg Care. 2007;23(11):829–31. 18007217 10.1097/PEC.0b013e31815a0667

[5] Gillman PK . Monoamine oxidase inhibitors, opioid analgesics and serotonin toxicity. Br J Anaesth. 2005;95(4):434–41. 16051647 10.1093/bja/aei210

[6] Okland T , Shirazi M , Rylander M , Holland J . A Case of Aggressive Psychosis in the Setting of Regular Dextromethorphan Abuse. Psychosomatics. 2016;57(6):655–6. 27814836 10.1016/j.psym.2016.06.002

[7] Smith MP , Lown M , Singh S , Ireland B , Hill AT , Linder JA , *et al* Acute Cough Due to Acute Bronchitis in Immunocompetent Adult Outpatients: CHEST Expert Panel Report. Chest. 2020;157(6):1256–65. 32092323 10.1016/j.chest.2020.01.044 PMC8173775

[8] The Japanese Respiratory Society . The JRS Guidelines for the Management of Cough and Sputum. 2019;1–188. Tokyo. [Japanese]

